# Temporal division of labor in an aphid social system

**DOI:** 10.1038/s41598-021-81006-z

**Published:** 2021-01-13

**Authors:** Harunobu Shibao, Mayako Kutsukake, Takema Fukatsu

**Affiliations:** 1 Graduate School of Life and Environmental Sciences, University of Tsukuba, Tsukuba, 305-8572 Japan; 2 Bioproduction Research Institute, National Institute of Advanced Industrial Science and Technology (AIST), Tsukuba, 305-8566 Japan; 3 Department of Biological Sciences, Graduate School of Science, The University of Tokyo, Tokyo, 113-0033 Japan

**Keywords:** Social evolution, Behavioural ecology, Evolutionary ecology, Entomology

## Abstract

Temporal division of labor, or age polyethism, in which altruistic caste individuals change their tasks with aging, is widely found in bees and ants (Hymenoptera) and also in other social insects. Here we report the discovery of elaborate age polyethism in a social aphid (Hemiptera). *Tuberaphis styraci* is a gall-forming aphid in which monomorphic first instar nymphs differentiate into normal nymphs and soldiers upon second instar molt. Soldiers neither grow nor reproduce but perform gall cleaning and colony defense. Using an artificial diet rearing system, we collected age-defined groups of soldiers and monitored their social behaviors. We observed that young soldiers tend to clean whereas old soldiers preferentially attack, thereby verifying age-dependent task switching from housekeeping to defense. Strategic sampling, age estimation and behavioral observation of soldiers from natural galls revealed that (1) young cleaning soldiers tend to inhabit upper gall regions with adult insects, (2) old attacking soldiers tend to be distributed in lower gall regions, particularly around the gall openings, and (3) the gall structure is linked to intra-nest movement, aging and task switching of soldiers in an adaptive manner. These results highlight an evolutionary parallelism comparable to the sophisticated temporal division of labor observed in honeybee colonies.

## Introduction

Social insects are characterized by reproductive, morphological and behavioral division of labor^1–3^. In the honeybee *Apis mellifera*, for example, a colony typically contains over 50,000 individuals, which consists of a single reproductive female called “queen” large in size, the majority of non-reproductive females called “workers” smaller in size, and some males called “drones”^4^. Only the queen lays eggs, whereas the workers perform a variety of social tasks such as nursing, glooming, nutrient exchange, nest building, guarding and foraging^5^. The monomorphic workers change their social tasks depending on their age, typically from nursing to foraging, which is called temporal division of labor or age polyethism^3,6,7^. The age polyethism in the honeybee is linked to the nest structure and the worker movement in the nest: newly-emerged workers at the central area of the comb perform nursing; as the newer workers emerge, the workers migrate to the peripheral area of the comb and conduct other inner-nest tasks; and finally the old workers go out of the nest and start collecting nectar and pollens^5,8^. While the sophisticated age polyethism in honeybee workers is the best-studied and well-known, some sorts of temporal division of labor have been reported from ants, termites and other social insects^1,9–14^. Theoretically and empirically, age polyethism entails the common pattern that safer tasks are performed at earlier life stages and hazardous tasks are postponed to later stages, by which life expectancy of altruistic individuals is maximized and thus the cost-effectiveness of the social investment is optimized^7,15,16^.

Some aphids are known to be social, which parthenogenetically produce female nymphs that perform altruistic tasks to the benefit of their colony mates at the expense of their own fitness^17–19^. Such nymphs are called “soldiers” because their primary social role is defense against natural enemies^20^, while some soldiers also perform other social tasks such as gall cleaning^21–23^, gatekeeping^24,25^ and gall repair^26–29^. In highly social aphids, soldiers are morphologically, physiologically and behaviorally differentiated from normal nymphs and unable to grow, constituting a sterile caste^18,30,31^.

*Tuberaphis styraci* (Hemiptera: Aphididae: Hormaphidinae) is a highly social aphid with morphological and reproductive division of labor. This species forms a large coral-shaped gall, which often exceeds 12 cm in diameter and contains over 20,000 insects in maturity, on the tree *Styrax obassia*. In the gall, adult females parthenogenetically produce monomorphic first instar nymphs, which differentiate into normal nymphs and soldiers upon second instar molt. While normal nymphs grow to adult and reproduce, soldiers neither grow nor reproduce but perform colony defense and housekeeping^23,32^. An artificial diet rearing system was developed for *T. styraci*^33^, which enabled experimental studies on the mechanisms underlying the density-dependent caste differentiation and regulation in the aphid social system^34–38^.

In this study, we demonstrate that a highly-organized temporal division of labor is operating in the gall colony of *T. styraci*. In the upper gall region, young soldiers preferentially perform gall cleaning; as aging proceeds, soldiers move down to the lower gall region; and old soldiers concentrate at the bottom gall region where exit holes exist and aggressively perform defense against enemies. These observations highlight an evolutionary parallelism in the relatively simple aphid society comparable to the sophisticated temporal division of labor observed in honeybee colonies.

## Materials and methods

### Insect life cycle and collection

 Galls of *T. styraci* are formed on *S. obassia* trees in mountainous areas of the mainland Japan^23,32^. In spring (typically from April to early May), small young galls contain a small number of aphids, wherein few soldiers are produced due to low aphid density. In early summer (typically from May to June), the galls grow quickly, in which the aphids rapidly increase in number and produce many soldiers due to elevated aphid density^34,35^. In summer to early autumn (typically from July to September), the mature galls are full of thousands of aphids with a considerable proportion of soldiers, which often exceed 60%^34,37^. In autumn, winged adult insects (called sexuparae) appear, fly away from the galls, and produce sexual females and males on the bark of *S. obassia*, and the galls eventually die^23,32^. The spring galls are too small to be detected and collected in the field. Hence, we regularly visited Shomaru, Saitama, Japan or Minakami, Gumma, Japan from May to August, where large galls of *T. styraci* harboring many aphids were collected from *S. obassia* trees.

### Insect rearing on artificial diet

The insects were harvested from the galls and maintained on artificial diet plates at 20 °C under a long day condition of 16 h light and 8 h dark in climate chambers as previously described^33^. The artificial diet plates are small plastic petri dishes (3.5 cm in diameter) on which filter-sterilized liquid diet is sandwiched between stretched Parafilm membranes, where the insects were kept and allowed to feed on the diet through the membrane. The diets were renewed every third day to prevent accumulation of honeydew and to minimize possible microbial contamination. The insects originating from the same set of galls were randomly divided into groups and used for experiments.

### Insect sampling, sorting and staging

The insects were sampled, sorted and staged as described below.

#### Nymphs and adults

Normal first, second, third and fourth instar nymphs, second instar soldiers, and adults were identified based on their size and morphology.

#### Young soldiers and old soldiers

For obtaining age-defined soldier populations, many first instar nymphs were kept on artificial diet plates and inspected everyday, and newly-molted soldiers were collected and transferred to new diet plates. Soldiers 10 days after molt and 20 days after second instar molt were defined as young soldiers and old soldiers, respectively.

#### Outer soldiers, inner soldiers and new soldiers

When field-collected galls were gently tapped, many soldiers came out of the gall openings, which were collected as “outer soldiers”. When no more soldiers came out after repeated tapping and collection, we broke the galls and sampled soldiers remaining inside. Some soldiers exhibited soft cuticle pale in color, which were collected as “new soldiers”. The remaining soldiers collected from the gall inside were categorized as “inner soldiers”.

#### Insects inhabiting upper gall region and lower gall region

From field-collected galls, upper gall pieces without openings (5 pieces per gall) and lower gall pieces with openings (5 pieces per gall) were sampled, from which all insects were collected, counted and identified.

### Estimation of remaining life span

Soldiers of interest were individually kept on artificial diet plates, inspected everyday, and monitored and recorded their life span.

### Assay of social behaviors

The insects were placed on artificial diet plates with colony wastes consisting of wax-coated honeydew globules, shed skins and corpses. For assaying cleaning behavior, the insects on an artificial diet plate were continuously observed for 1 h under a dissection microscope. When an insect continuously pushed a colony waste for 5 s or longer, the insect was regarded as performing cleaning behavior. For assaying attacking behavior, the insects on an artificial diet plate were stimulated with a fine brush. When an insect reacted to the brush, climbed onto it, and attempted to sting it with the stylet within 10 s, the insect was regarded as performing attacking behavior. When the same insects were serially assayed for their social behaviors, the cleaning behavior was monitored first, and then the attacking behavior was inspected.

## Results and discussion

### Assay of soldier’s social behaviors on artificial diet rearing system

**Figure 1:**
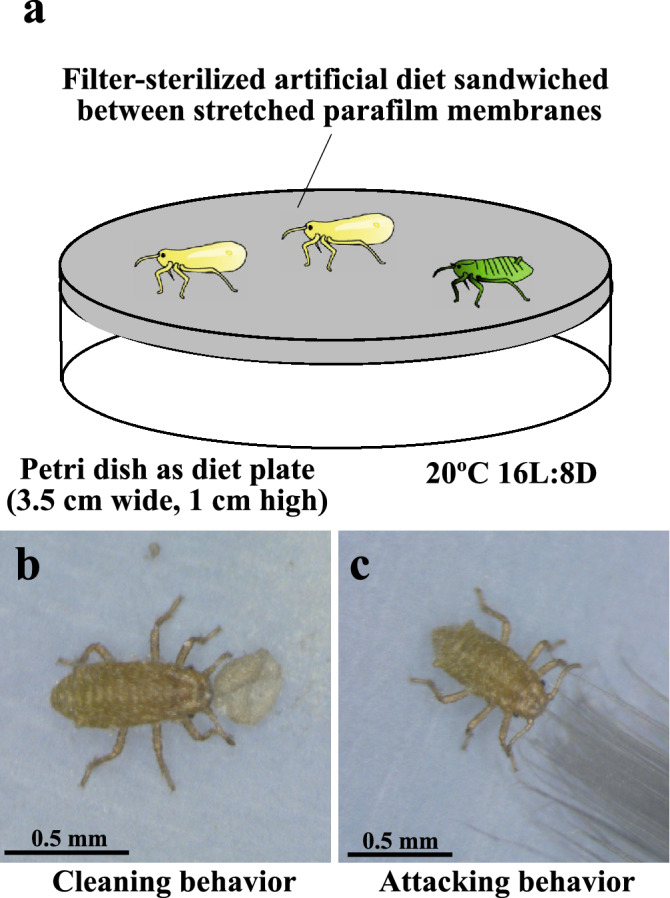
(**a**) Artificial diet rearing system for *T. styraci* after Shibao et al.^33^. (**b**,**c**) Behavioral assays for cleaning behavior (**b**) and attacking behavior (**c**) on artificial diet plates.

In natural galls of *T. styraci*, soldiers exhibit two distinct social behaviors: cleaning behavior to push colony wastes (wax-coated honeydew globules, shed skins, corpses, etc.) with the head continuously, finally disposing them through openings located at the bottom of the gall; and attacking behavior to cling to enemies and sting them with the stylet, finally paralyzing or killing the intruders^23,32^. We found that, since these social behaviors are so stereotypic, soldiers perform the same behaviors even under the very unnatural environment on the artificial diet plates (Fig. 1a): when encountering a honeydew globule, soldiers push it continuously (Fig. 1b); when stimulated with a fine brush, soldiers cling to it and attempt to sting it with the stylet (Fig. 1c). In this way, we established simple experimental procedures for assaying the social behaviors of soldiers and other colony members of *T. styraci* in the laboratory.

### Soldiers perform cleaning and attacking behaviors on artificial diet rearing system

**Figure 2:**
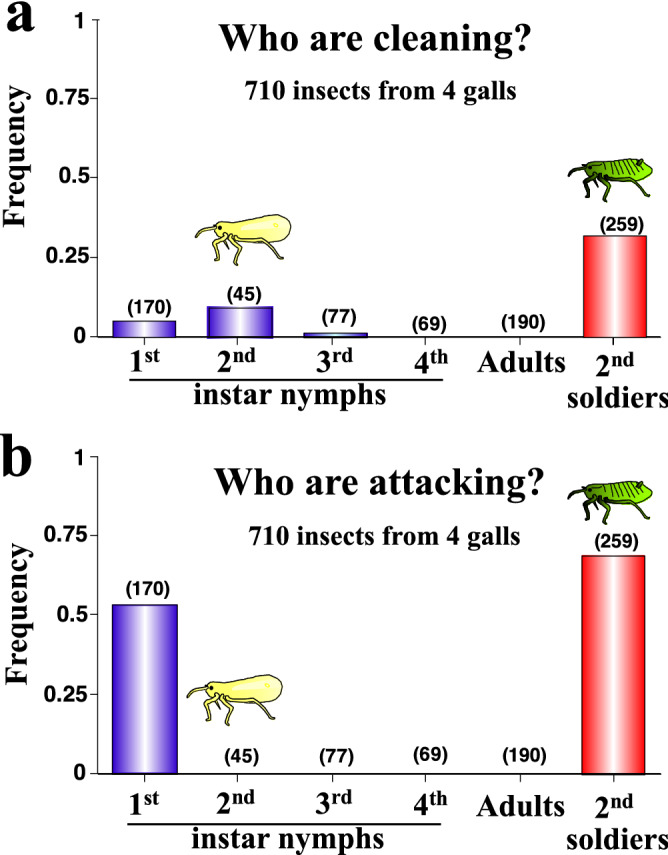
Social behaviors performed by gall inhabitants of *T. styraci* on artificial diet plates. (**a**) Frequency of cleaning behavior. A statistically significant difference was detected between soldiers and second instar non-soldier nymphs (Chi-square test; *P* < 0.01). (**b**) Frequency of attacking behavior. Statistically significant differences were identified between soldiers and second instar non-soldier nymphs (Chi-square test; *P* < 0.0001) and between soldiers and first instar nymphs (Chi-square test; *P* < 0.01). In total, 710 insects from 4 galls, which contained first, second, third and fourth instar nymphs, unwinged adults, and second instar soldiers, were subjected to behavioral observations.

We collected soldiers, nymphs and adults of *T. styraci* from natural galls, and observed their social behaviors on the artificial diet plates. The cleaning behavior was mainly performed by soldiers, whereas first, second and third instar nymphs also exhibited the cleaning behavior though less frequently (Fig. 2a). The attacking behavior was performed only by soldiers and first instar nymphs at considerably high frequencies (Fig. 2b). Here, the attacking first instar nymphs are probably destined to become soldiers upon second instar molt^39^. These results indicate that soldiers mainly perform cleaning and attacking behaviors in *T. styraci* as previously reported^23,32^.

### Young soldiers clean whereas old soldiers attack

**Figure 3:**
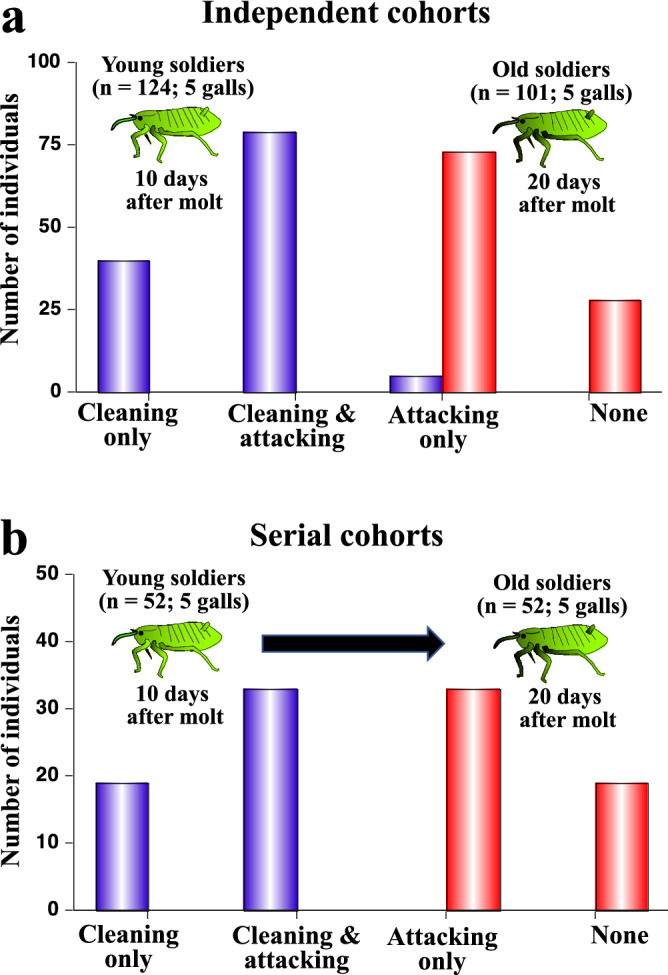
Social behaviors performed by young soldiers and old soldiers of *T. styaci* reared on artificial diet plates. (**a**) Observations of independent cohorts, in which 124 young soldiers (10 days after second instar molt) from 5 galls and 101 old soldiers (20 days after second instar molt) from 5 galls were independently collected and subjected to behavioral observations. Difference in frequencies of social behaviors between young soldiers and old soldiers was statistically significant (Chi-square test; *P* < 0.0001). (**b**) Observations of serial cohorts, in which 52 young soldiers (10 days after second instar molt) from 5 galls were collected and subjected to behavioral observations, and 10 days later, the same 52 soldiers (20 days after second instar molt) were subjected to behavioral observations again. Difference in frequencies of social behaviors between young soldiers and old soldiers was statistically significant (Chi-square test; *P* < 0.0001).

By keeping many first instar nymphs on the artificial diet plates, inspecting them every day, and transferring newly-molted soldiers to new diet plates, we collected age-defined soldier populations of *T. styraci*. When 10-day-old soldier populations were observed for their social behaviors, they performed either cleaning only or both cleaning and attacking (Fig. 3a). By contrast, when 20-day-old soldier populations were examined, they performed either attacking only or no social behaviors (Fig. 3a). When the same soldier populations were examined for their social behaviors 10 days after molting and 20 days after molting serially, similar behavioral patterns were observed (Fig. 3b).

### Temporal division of labor in aphid soldiers observed in laboratory colonies

These results strongly suggest that soldiers of *T. styraci* exhibit temporal division of labor, or age polyethism, in which young soldiers preferentially perform cleaning behavior for colony maintenance and old soldiers mainly exhibit attacking behavior for colony defense against enemies. It is notable that these behavioral patterns look similar to those known for other social insects. For example, in honeybees, young workers perform nursing for taking care of larvae within the nest, whereas old workers are engaged in foraging for collecting nectar and pollens outside the nest^5,6,8^. Meanwhile, we should be cautious in interpreting these results obtained under a quite unnatural condition for the aphid colonies maintained on the artificial diet plates. Are the same behavioral patterns observed in natural aphid galls?

### Estimating age of soldiers collected from natural aphid galls

**Figure 4:**
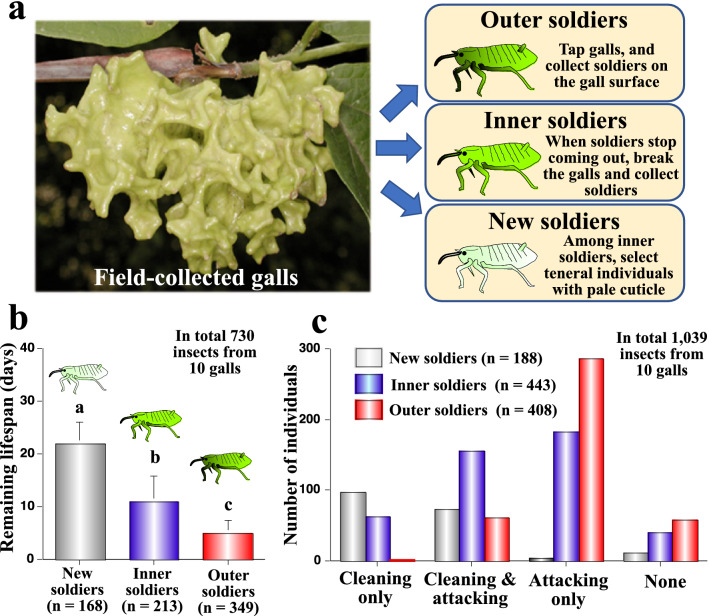
(**a**) A coral-shaped gall of *T. styaci*, and sampling procedures for outer soldiers, inner soldiers and new soldiers from field-collected galls. (**b**) Remaining life span of new soldiers, inner soldiers and outer soldiers measured on artificial diet plates. Different alphabetical letters (**a**–**c**) indicate statistically significant differences (Log-rank test; *P* < 0.001) (**c**) Social behaviors performed by new soldiers, inner soldiers and outer soldiers on artificial diet plates. Statistically significant differences were found in frequencies of social behaviors among new soldiers, inner soldiers, and outer soldiers (pairwise Chi-square tests; all *P* < 0.0001). In total, 1,039 soldiers from 10 galls, which consist of 188 new soldiers, 443 inner soldiers and 408 outer soldiers, were examined.

When we collect a natural gall of *T. styraci* from the host plant twig, this stimulus elicits conspicuous behaviors of soldiers: many soldiers come out from the openings at the bottom of the gall, crawl around on the gall surface, and exhibit attacking behavior when encountering other insects. After a while, soldiers calm down and retreat to the gall inside, but when we lightly tap the gall surface, soldiers come out again and repeat the same behaviors. By tapping the gall and collecting soldiers from the gall surface, we sampled “outer soldiers”. When no more soldiers came out after repeated tapping and collection, we broke the gall and collected soldiers remaining inside the gall. Some soldiers exhibited soft cuticle pale in color, evidently being just after molting, which were sampled as “new soldiers”. The remaining soldiers collected from the gall inside were categorized as “inner soldiers” (Fig. 4a). By rearing these insects on artificial diet plates individually, we measured remaining lifespan of the individual field-collected soldiers. On average, new soldiers, inner soldiers and outer soldiers survived for 21 days, 10 days and 5 days, respectively (Fig. 4b). These results indicate that new soldiers, inner soldiers and outer soldiers actually represent newly-molted soldiers, young soldiers and old soldiers, respectively. It is also estimated that the soldier’s physiological lifespan may be around three weeks.

### Young soldiers clean whereas old soldiers attack just after collection from natural galls

Just after collection from natural galls and before measurement of remaining life span, we inspected social behaviors of new soldiers, inner soldiers and outer soldiers individually on the artificial diet plates. The results were clear: new soldiers preferentially exhibited cleaning behavior; old soldiers mainly performed attacking behavior; and inner soldiers were intermediate (Fig. 4c).

### Temporal division of labor in aphid soldiers collected from natural galls

These results confirm that, in natural colonies of *T. styraci*, soldiers exhibit temporal division of labor in which young soldiers preferentially perform cleaning behavior for colony maintenance and old soldiers mainly exhibit attacking behavior for colony defense against enemies. Thus far, several studies have pointed out notable spatial and temporal patterns of soldier distribution in social aphid colonies^40–44^. To our knowledge, however, this study is the first to report an evident case of age polyethism among eusocial species in the Hemiptera. Conceivably, as observed in honeybee colonies^5,6,8^, young soldiers with long expected lifespan are engaged in safer housekeeping tasks, whereas old soldiers with short expected lifespan are allocated to risky defensive tasks, by which the aphid colonies efficiently utilize the social investment in the sterile soldier caste^7,15,16^.

### Young cleaning soldiers deep in the upper gall region and old aggressive soldiers in the lower gall periphery

**Figure 5:**
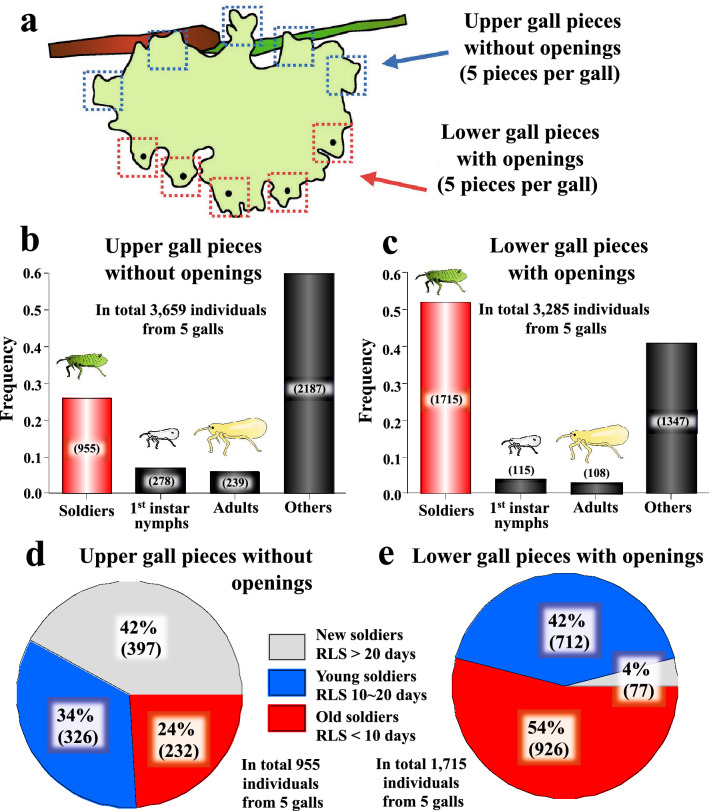
(**a**) Sampling procedures for gall inhabitants of *T. styraci* from upper gall pieces and lower gall pieces. (**b**, **c**) Compositions of gall inhabitants in upper gall pieces (**b**) and lower gall pieces (**c**). The insects were categorized into soldiers, first instar nymphs, adults, and others based on their morphological traits. A statistically significant difference was found in frequencies of four aphid classes between upper gall pieces and lower gall pieces (Chi-square test; *P* < 0.0001). (**d**, **e**) Age compositions of soldiers collected from upper gall pieces (**d**) and lower gall pieces (**e**). The remaining life span (RLS) of soldiers was individually evaluated on artificial diet plates, by which they were categorized into the following age classes: new soldiers, RLS > 20 days; young soldiers, RLS 10–20 days; old soldiers, RLS < 10 days. A statistically significant difference was found in frequencies of three age classes between upper gall pieces and lower gall pieces (Chi-square test; *P* < 0.0001).

In honeybees, temporal division of labor in workers entail their gradual spatial movement across the nest structure: namely, young workers emerge from brood combs located at the central nest and perform nursing for taking care of larvae there; middle-aged workers move to the peripheral nest areas and undertake a variety of colony tasks; and old workers go out of the nest and perform foraging for collecting nectar and pollens^5,8^. In an attempt to examine whether such patterns are also observed in natural colonies of the social aphid, we sampled upper gall pieces without openings (5 pieces per gall) and lower gall pieces with openings (5 pieces per gall), from which all insects were collected, counted and identified (Fig. 5a). The colony composition data revealed that soldiers tended to be found more frequently in the lower gall pieces than in the upper gall pieces, whereas first instar nymphs and adults tended to inhabit more frequently in the upper gall pieces than in the lower gall pieces (Fig. 5b, c). When the collected soldiers were individually assayed for their remaining lifespan on the artificial diet plates, it turned out that the upper gall pieces contained 42% new soldiers, 34% young soldiers and 24% old soldiers, whereas the lower gall pieces harbored 4% new soldiers, 42% young soldiers and 54% old soldiers (Fig. 5d, e). These results indicate that reproductive adult females, newborn nymphs and newly-molted soldiers tend to inhabit the upper gall region while old soldiers are preferentially found in the lower gall region, suggesting aging-related downward movement of soldiers in natural galls of *T. styraci.*

### Relationship between gall structure, aging of soldiers, and temporal division of labor

**Figure 6:**
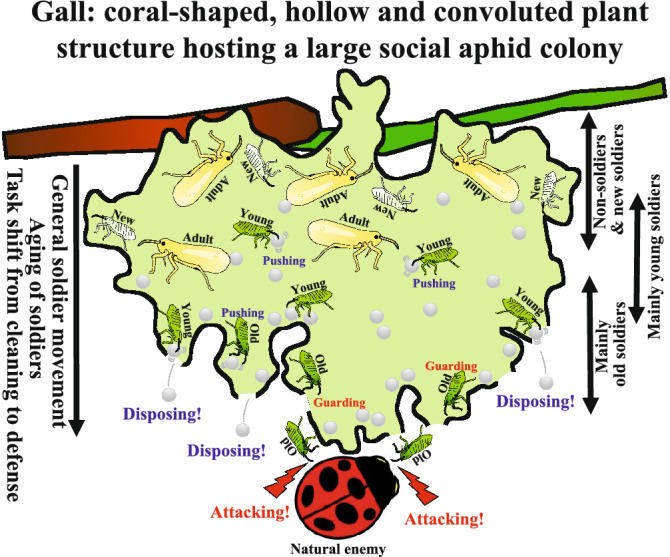
Graphical presentation of age polyethism in natural gall colony of *T. styraci*, highlighting the relevance of gall structure to production, aging and movement of soldiers.

All these results taken together, we propose a hypothetical model as to how gall structure, aging of soldiers, and temporal division of labor are related to each other and, as a whole, integrated into adaptive consequences for the natural social aphid colonies (Fig. 6). Adult aphids mainly inhabit deep in the gall cavity, typically in the safe upper gall region, where first instar nymphs are produced and some of them become second instar soldiers in a density-dependent manner^35,38^. Young soldiers preferentially perform cleaning behavior by pushing honeydew globules, shed skins and aphid corpuses, by which the colony wastes are moved down to the lower gall region. Owing to the continuous cleaning behavior in combination with the action of gravity, the soldiers gradually move down to the lower gall region, with switching their behavior from cleaning to defense. As a consequence, old aggressive soldiers are concentrated in the lower gall region where the aphid colony is vulnerable to enemy’s intrusion through the openings for waste disposal. In this way, optimal spatial allocation of cleaning young soldiers and attacking old soldiers is realized through temporal task switching of soldiers and structural configuration of the aphid galls.

## Conclusion and perspective

In comparison with the spectacular complexity and elaboration of social systems in bees, ants and termites^1,2,9,45^, the aphid social systems look simple and primitive—characterized by small colony size, simple caste system, stereotypic and non-flexible social behaviors, absence of kin recognition, absence of nutrient exchange between colony mates, etc.^17–19,24,46–48^. Notwithstanding this, our previous studies revealed that the social aphid *T. styraci* has evolved an appreciably complex social system that entails formation of structurally complex plant-made nest of coral-shaped gall^38,49^, relatively large colony with over 20,000 insects^35,37^, density-dependent regulation mechanisms for soldier caste differentiation^34–38^, and evolution of soldier-specific venomous protease^39,50^. In this study, we uncovered another layer of elaborate mechanism underpinning the aphid social system, age polyethism, that is, though simpler, comparable to the sophisticated temporal division of labor observed in honeybee colonies. This finding highlights that the level of social complexity in aphids may be considerably higher than previously envisioned.

## References

[1] Wilson EO (1971). The Insect Societies.

[2] Wilson EO (1975). Sociobiology: The New Synthesis.

[3] Oster GF, Wilson EO (1978). Caste and Ecology in the Social Insects.

[4] Seeley TD (1985). Honeybee Ecology: A Study of Adaptation in Social Life.

[5] Seeley TD (1982). Adaptive significance of the age polyethism schedule in honeybee colonies. Behav. Ecol. Sociobiol..

[6] Robinson GE (1992). Regulation of division of labor in insect societies. Annu. Rev. Entomol..

[7] Beshers SN, Fewell JH (2001). Models of division of labor in social insects. Annu. Rev. Entomol..

[8] Johnson BR (2008). Within-nest temporal polyethism in the honey bee. Behav. Ecol. Sociobiol..

[9] Hölldobler B, Wilson EO (1990). The Ants.

[10] Crosland MWJ, Lok CM, Wong TC, Shakarad M, Traniello JFA (1997). Division of labour in a lower termite: The majority of tasks are performed by older workers. Anim. Behav..

[11] Hinze B, Leuthold RH (1999). Age related polyethism and activity rhythms in the nest of the termite *Macrotermes bellicosus* (Isoptera, Termitidae). Insect. Soc..

[12] Cameron SA (1989). Temporal patterns of division of labor among workers in the primitively eusocial bumble bee, *Bombus griseocoffis* (Hymenoptera: Apidae). Ethology.

[13] Naug D, Gadagkar R (1998). The role of age in temporal polyethism in a primitively eusocial wasp. Behav. Ecol. Sociobiol..

[14] Biedermann PHW, Taborsky M (2011). Larval helpers and age polyethism in ambrosia beetles. Proc. Natl. Acad. Sci. USA.

[15] Wakano JN, Nakata K, Yamamura N (1998). Dynamic model of optimal age polyethism in social insects under stable and fluctuating environments. J. Theor. Biol..

[16] Duarte A, Weissing FJ, Pen I, Keller L (2011). An evolutionary perspective on self-organized division of labor in social insects. Annu. Rev. Ecol. Evol. Syst..

[17] Stern DL, Foster WA (1996). The evolution of soldiers in aphids. Biol. Rev..

[18] Aoki S, Kurosu U (2010). A review of the biology of Cerataphidini (Hemiptera, Aphididae, Hormaphidinae), focusing mainly on their life cycles, gall formation, and soldiers. Psyche.

[19] Abbot P, Tooker J, Lawson SP (2018). Chemical ecology and sociality in aphids: Opportunities and directions. J. Chem. Ecol..

[20] Aoki S (1977). *Colophina clematis* (Homoptera, Pemphigidae), an aphid species with" soldiers". Kontyu.

[21] Aoki S, Kurosu U (1989). Gall cleaning by the aphid *Hormaphis betulae*. J. Ethol..

[22] Benton TG, Foster WA (1992). Altruistic housekeeping in a social aphid. Proc. R. Soc. B.

[23] Aoki S, Kurosu U (1989). Soldiers of *Astegopteryx styraci* (Homoptera, Aphidoidea) clean their gall. Jpn. J. Entomol..

[24] Aoki S, Kurosu U, Stern DL (1991). Aphid soldiers discriminate between soldiers and non-soldiers, rather than between kin and non-kin *Ceratoglyphina bambusae*. Anim. Behav..

[25] Kurosu U, Narukawa J, Buranapanichpan S, Aoki S (2006). Head-plug defense in a gall aphid. Insect. Soc..

[26] Kurosu U, Aoki S, Fukatsu T (2003). Self-sacrificing gall repair by aphid nymphs. Proc. R. Soc. B.

[27] Pike N, Foster W (2004). Fortress repair in the social aphid species *Pemphigus spyrothecae*. Anim. Behav..

[28] Kutsukake M, Shibao H, Uematsu K, Fukatsu T (2009). Scab formation and wound healing of plant tissue by soldier aphid. Proc. R. Soc. B.

[29] Kutsukake M, Moriyama M, Shigenobu S, Meng XY, Nikoh N, Noda C, Kobayashi S, Fukatsu T (2019). Exaggeration and cooption of innate immunity for social defense. Proc. Natl. Acad. Sci. USA.

[30] Aoki S, Ito Y, Brown JL, Kikkawa J (1987). Evolution of sterile soldiers in aphids. Animal Societies: Theories andFacts.

[31] Aoki S, Kurosu U, Starr CK (2020). Social aphids. Encyclopedia of Social Insects.

[32] Aoki S, Kurosu U (1990). Biennial galls of the aphid *Astegopteryx styraci* on a temperate deciduous tree* Styrax obassia*. Acta Phytopathol. Entomol. Hung..

[33] Shibao H, Kutsukake M, Lee J, Fukatsu T (2002). Maintenance of soldier-producing aphids on an artificial diet. J. Insect Physiol..

[34] Shibao H, Lee JM, Kutsukake M, Fukatsu T (2003). Aphid soldier differentiation: density acts on both embryos and newborn nymphs. Naturwissenschaften.

[35] Shibao H, Kutsukake M, Fukatsu T (2004). Density triggers soldier production in a social aphid. Proc. R. Soc. B.

[36] Shibao H, Kutsukake M, Fukatsu T (2004). The proximate cue of density-dependent soldier production in a social aphid. J. Insect Physiol..

[37] Shibao H, Kutsukake M, Fukatsu T (2004). Density-dependent induction and suppression of soldier differentiation in an aphid social system. J. Insect Physiol..

[38] Shibao H, Kutsukake M, Matsuyama S, Fukatsu T, Shimada M (2010). Mechanisms regulating caste differentiation in an aphid social system. Commun. Integr. Biol..

[39] Kutsukake M, Shibao H, Nikoh N, Morioka M, Tamura T, Hoshino T, Ohgiya S, Fukatsu T (2004). Venomous protease of aphid soldier for colony defense. Proc. Natl. Acad. Sci. USA.

[40] Stern DL, Aoki S, Kurosu U (1994). A test of geometric hypotheses for soldier investment patterns in the gall producing tropical aphid *Cerataphis fransseni* (Homoptera, Hormaphididae). Insect. Soc..

[41] Pike N, Braendle C, Foster WA (2004). Seasonal extension of the soldier instar as a route to increased defence investment in the social aphid *Pemphigus spyrothecae*. Ecol. Entomol..

[42] Pike N (2007). Specialised placement of morphs within the gall of the social aphid *Pemphigus spyrothecae*. BMC Evol. Biol..

[43] Uematsu K, Kutsukake M, Fukatsu T, Shimada M, Shibao H (2010). Altruistic colony defense by menopausal female insects. Curr. Biol..

[44] Uematsu K, Shimada M, Shibao H (2013). Juveniles and the elderly defend, the middle-aged escape: division of labour in a social aphid. Biol. Let..

[45] Abe T, Bignell DE, Higashi M, Abe Y (2000). Termites: Evolution, Sociality, Symbioses, Ecology.

[46] Shibao H (1999). Lack of kin discrimination in the eusocial aphid *Pseudoregma bambucicola* (Homoptera: Aphididae). J. Ethol..

[47] Abbot P, Withgott JH, Moran NA (2001). Genetic conflict and conditional altruism in social aphid colonies. Proc. Natl. Acad. Sci. USA.

[48] Abbot P, Chhatre V (2007). Kin structure provides no explanation for intruders in social aphids. Mol. Ecol..

[49] Kutsukake M, Meng XY, Katayama N, Nikoh N, Shibao H, Fukatsu T (2012). An insect-induced novel plant phenotype for sustaining social life in a closed system. Nat. Commun..

[50] Kutsukake M, Nikoh N, Shibao H, Rispe C, Simon JC, Fukatsu T (2008). Evolution of soldier-specific venomous protease in social aphids. Mol. Biol. Evol..

